# Cannabis-based extract for managing pain in dogs with osteoarthritis: efficacy and safety assessment

**DOI:** 10.3389/fphar.2025.1539704

**Published:** 2025-11-24

**Authors:** Neide Maria Griebeler, Ricardo Penayo Cremonese, Yasmin Rafaela Fakih Correa, Priscila Romero Mazzini Pereira, Amanda Furjan Rial, Emanuela Leite, Maria Victoria Luz Gonçalves, Luiz Renato Marques das Almas, Nedice Borges Cardoso, Fernando Cezar-dos-Santos, Aline Theodoro Toci, Andrés Mojoli Le-Quesne, Francisney Pinto Nascimento

**Affiliations:** 1 Laboratory of Cannabis and Psychedelics - LCP, Universidade Federal da Integração Latino Americana (UNILA), Foz do Iguaçu, Brazil; 2 POP-VET Veterinary Hospital, Foz do Iguaçu, Brazil; 3 Associação Santa Cannabis, Florianópolis, Brazil; 4 Environmental and Food Interdisciplinary Studies Laboratory - LEIMAA, Universidade Federal da Integração Latino Americana (UNILA), Foz do Iguaçu, Brazil

**Keywords:** adverse events, cannabinoids, canine, efficacy, osteoarthritis, quality of life, safety

## Abstract

**Introduction:**

Canine osteoarthritis (OA) is a prevalent disease characterized by progressive joint degeneration, pain, and impaired mobility. In older dogs, OA affects approximately 80% of the population. Current pharmacological treatments are often limited in efficacy and may cause significant adverse effects. In this context, cannabinoids have emerged as a promising therapeutic alternative.

**Methods:**

This double-blind, randomized study aimed to evaluate the efficacy and safety of a full-spectrum cannabis extract in managing OA-related pain and dysfunction in dogs. Seventeen dogs with OA were randomly assigned to receive either the cannabis extract (containing measured amounts of CBD, THC, CBG, CBC, and other cannabinoids) or a placebo for 90 days.

**Results:**

Although the cannabis treatment did not significantly reduce pain levels according to the Helsinki Chronic Pain Index (HCPI) [F(1,14) = 0.001, p = 0.981, η^2^ = 0.000], a reduction of 2.4 points in HCPI scores compared to the placebo was observed at 90 days. Additionally, the treatment proved to be entirely safe, with no significant adverse effects reported. The few mild side effects observed resolved spontaneously within 24 h. Biomarker analysis revealed no significant differences between the cannabis and placebo groups.

**Conclusion:**

Our findings suggest that full-spectrum cannabis extract containing CBD and THC is safe for use in dogs for up to 90 days. However, further research is needed to determine the optimal doses and formulations required to effectively alleviate OA-induced pain.

## 1 Introduction

Canine osteoarthritis (OA) is a common degenerative disease affecting the knee and hip joints, characterized by articular cartilage loss, osteophyte formation, and synovial inflammation (Dieppe and Lohmander, 2005). The primary clinical sign is pain, accompanied by joint stiffness, lameness, and reluctance to engage in daily activities, all of which significantly impair the animal’s quality of life (Brown et al., 2007; Gildea et al., 2024). Large dog breeds with higher body weight are at increased risk of developing OA (Anderson et al., 2020). Reports indicate that approximately 20% of dogs over 1 year of age are affected by OA, while this prevalence rises to 80% in dogs over 8 years old (Johnston, 1997; Bhathal et al., 2017).

Non-steroidal anti-inflammatory drugs (NSAIDs) are the first-line pharmacological treatment for OA pain, though their efficacy is often limited (Vaughan-Scott and Taylor, 1997; Harper, 2017). Additionally, NSAIDs can induce adverse effects involving the gastrointestinal, renal, and hepatic systems, with gastrointestinal complications being the most common reason for treatment discontinuation (Papich, 2008; Khan and McLean, 2012). Thus, there is a clear need to explore new OA treatments with improved efficacy and safety profiles. In this context, phytocannabinoids have emerged as a promising therapeutic option.

Recent evidence from OA animal models supports the analgesic potential of phytocannabinoids (Philpott et al., 2017; O’Brien and McDougall, 2018). Among the hundreds of phytocannabinoids, the most abundant are cannabidiol (CBD) and Δ9-tetrahydrocannabinol (THC), both derived from *Cannabis sativa*. These compounds interact with the endocannabinoid system (ECS), specifically targeting CB1 and CB2 receptors. Since these receptors are distributed throughout peripheral and central pain pathways (McDougall and Linton, 2012), and since OA leads to increased ECS activation (Fu et al., 2018), modulating the ECS with CBD, THC, or other cannabinoids could provide peripheral and central pain relief, making them a viable therapeutic option for OA (O’Brien and McDougall, 2018; Patikorn et al., 2023). Thus, we hypothesized that a full-spectrum cannabis extract with known concentrations of CBD, THC, CBC, and CBG could reduce pain and improve the quality of life in dogs with osteoarthritis. Then, we aimed to evaluate the therapeutic potential of a full-spectrum cannabis extract (containing CBD, THC, and other cannabinoids) in dogs diagnosed with OA. The study was designed as a double-blind, placebo-controlled, randomized trial with a 90-day follow-up period.

## 2 Materials and methods

### 2.1 Location of research and ethics statements

The study was conducted at the Medical Cannabis and Psychedelic Science Laboratory of the Universidade Federal da Integração Latino-Americana (UNILA) and at the PopVet Veterinary Hospital, both located in the city of Foz do Iguaçu, Brazil. Ethical approval for the study was provided by UNILA’s Animal Ethics Committee under protocol number 1/2023.

### 2.2 Cannabis extraction and quantification of cannabinoids

**FIGURE 1:**
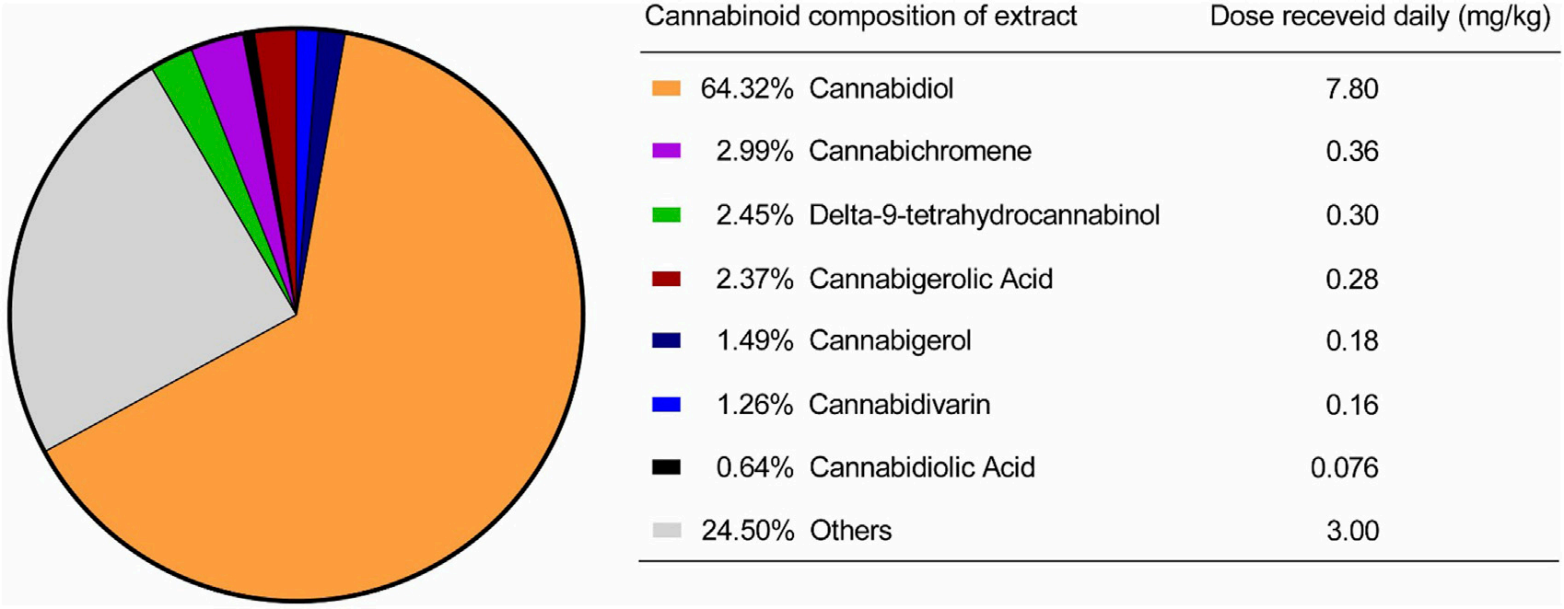
Cannabinoids present in the extract provided by the Santa Cannabis Association.

For the cannabinoid extraction process, the inflorescences were treated as described by Salazar-Bermeo et al. (2023). After drying in a controlled environment, the material was ground and subjected to extraction with 99.8% ethanol (Sigma, United States) at −30 °C. The solvent was removed using a rotary evaporator (Mylabor, Brazil) under reduced pressure and low temperature, yielding the raw ethanolic extract (full spectrum). The cannabinoid profile and concentration analysis was performed by high-performance liquid chromatography (HPLC–Shimadzu, Japan) using a C18 column and a solvent flow rate of 0.5 mL/min, with a gradient between solvents A (0.1% formic acid in water) and B (0.1% formic acid in acetonitrile). The gradient followed the program: 0–8 min, 65% solvent B; 8–12 min, 65%–95% B; and 12–13 min, 95% B. The injection volume was 5 μL, and the cannabinoids were monitored at 214 nm, as described by Moreno-Chamba et al. (2024). Quantification was performed using an external calibration curve with five acidic cannabinoids: tetrahydrocannabinolic acid (THCA), cannabidiolic acid (CBDA), tetrahydrocannabivarinic acid (THCVA), cannabidivarinic acid (CBDVA), and cannabigerolic acid (CBGA), as well as eight neutral cannabinoids: delta-9-tetrahydrocannabinol (Δ9-THC), delta-8-tetrahydrocannabinol (Δ8-THC), cannabidiol (CBD), cannabinol (CBN), cannabichromene (CBC), cannabigerol (CBG), tetrahydrocannabivarin (THCV), and cannabidivarin (CBDV). The concentration of each phytocannabinoid present in the extract is shown in Figure 1. The full-spectrum cannabis extract used in this study was provided by the Santa Cannabis Association in Florianópolis, Brazil.

### 2.3 Study design

The study was conducted as a double-blind, randomized, placebo-controlled clinical trial. A total of 33 dogs diagnosed with osteoarthritis were initially recruited. Of these, 16 could not be randomized due to not meeting inclusion criteria, death or other logistical reasons. The remaining 17 dogs were randomly allocated in a 1:1 ratio, resulting in 8 assigned to the placebo group, which received medium chain triglycerides (MCT), and 9 assigned to the treatment group, which received a full-spectrum cannabis extract diluted in MCT. Ultimately, 7 animals in each arm completed the study.

### 2.4 Participant selection, randomization, treatment administration, and blinding

Inclusion criteria required a diagnosis of hip osteoarthritis confirmed by radiographic examination, osteoarthritis-related pain, and that animals be at least 1 year old. Additionally, owners had to voluntarily consent to their dog’s participation by signing the Free and Informed Consent Form. Dogs were excluded if they were diagnosed with neurological disorders, kidney disease, severe liver failure, ehrlichiosis infection, or if they had used dietary supplements, NSAIDs, gabapentin, or other analgesics within the past 4 months. To ensure the presence of clinically meaningful chronic pain, only dogs with a Helsinki Chronic Pain Index (HCPI) score of 11 or higher at baseline were eligible for inclusion.

Following this initial phase, dogs eligible for the study were randomized using the random.org system in a 1:1 ratio, taking into account the animals’ pain levels. Animal weights were obtained by direct measurement with a calibrated digital scale. Each animal in the treatment group received by oral route a full spectrum cannabis oil twice daily, totaling a daily dose of 7.8 mg/kg of CBD, 0.30 mg/kg of THC, 0.36 mg/kg of CBC, 0.28 mg/kg of CBGA, and 0.18 mg/kg of CBG, in addition to other cannabinoids and components in smaller proportions. The dose selection for this trial was primarily based on a THC dosage of 0.30 mg/kg, guided by the extensive clinical experience of this study’s veterinary physician and our hospital team, albeit without prior scientific publication. Meanwhile, animals in the placebo group were given a volume-matched solution containing only MCT oil, with no cannabis component. All treatments were administered orally every 12 h for 90 days.

To maintain the double-blind design, both owners and veterinarians responsible for clinical evaluations were blinded to treatment allocation. The cannabis extract and the placebo (MCT oil) were dispensed in identical bottles with matching droppers to ensure similar appearance and handling. Randomization codes were generated and managed peby a researcher not involved in clinical assessments or data analysis, who also prepared and labeled the treatments according to the allocation sequence. Treatment codes were securely stored and disclosed only after all analyses were completed. This procedure ensured that clinical assessments, owner-reported outcomes, and data evaluations were conducted without knowledge of group assignments.

### 2.5 Clinical assessment

#### 2.5.1 Helsinki chronic pain index

The Helsinki Chronic Pain Scale was applied to the owner by our veterinarian to assess pain in canine osteoarthritis (Hielm-Björkman et al., 2003). The scale consists of 11 questions focused on the dog’s general activity. Owners completed the scale by rating each item on a visual analog scale, with endpoints representing the extremes of each characteristic.

#### 2.5.2 CBPI questionnaire

Pain and quality of life in dogs were evaluated using the CBPI questionnaire, administered to the owners by the veterinarian. (Brown et al., 2008). Briefly, this scale consists of three sections: (1) pain severity, (2) pain interference with daily activities, and (3) quality of life. The pain severity section measures the dog pain level. The pain interference section assesses how pain affects the dog’s general activity, enjoyment of life, ability to rise, and mobility. The quality-of-life section evaluates the owner’s overall perception of the dog’s wellbeing (Brown et al., 2007).

#### 2.5.3 Veterinary clinical assessment (VCA)

The veterinary physician evaluated the severity of clinical signs was evaluated by the Veterinary Physician using an established ordinal scoring system (McCarthy et al., 2007), which rated lameness, joint mobility, pain on palpation, and weight-bearing on a scale from 1 (least affected) to 5 (most affected).

### 2.6 Safety assessments

#### 2.6.1 Adverse effects assessment

Owners were also instructed to complete an adverse event register, recording the date and nature of any incidents during the study. The incidence and types of adverse events were systematically compared between the control and cannabis treatment groups at all time points (Brioschi et al., 2020; Vaughn et al., 2020). The adverse event log provided to owners consisted of a standardized form designed to record the date, time, description, and duration of any observed clinical signs. The form included a checklist of common potential adverse effects such as diarrhea, vomiting, lethargy, agitation, salivation, appetite changes, and behavioral alterations. Owners were instructed to monitor their animals closely, particularly within the first hour after each administration, and to record any signs regardless of perceived severity. Contact information for the responsible veterinarian was included for reporting unexpected or severe reactions. All completed logs were reviewed at each follow-up visit to ensure consistent documentation of adverse events.

#### 2.6.2 Biochemical safety assessment

Additionally, serum biochemical parameters, including liver and kidney function tests, were analyzed from blood samples. Serum concentrations of alanine aminotransferase (ALT), aspartate aminotransferase (AST), alkaline phosphatase (ALP), gamma-glutamyl transferase (GGT), albumin, and total bilirubin were quantified to assess liver integrity and function. Concurrently, renal function was evaluated by measuring serum creatinine and urea levels. Further, levels of total protein, globulin, cholesterol, triglycerides and glucose were also measured. All animals were also tested for leishmaniasis and vianaehrlichiosis infections using the ELISA method (Viana et al., 2018; Zorzo et al., 2023). These data were collected at baseline, as well as at 30 and 90 days post-treatment.

Blood samples for the analysis of these analytes and biomarkers were collected immediately prior to the initiation of cannabinoid treatment (baseline) and upon completion of the clinical trial (day 90). All samples were stored at −80 °C in our veterinary hospital facility and processed in a single batch at the end of the study. All assays were performed according to standardized protocols in a certified clinical laboratory.

#### 2.6.3 Glasgow coma scale

Potential neurological adverse effects, such as changes in the level of consciousness, motor function, brainstem function, or oculocephalic reflexes, were assessed using the Glasgow Coma Scale (Platt et al., 2001). In this scale, lower scores indicate more severe neurological impairment.

### 2.7 Statistical analysis

The primary efficacy analysis was conducted according to a modified intention-to-treat principle, incorporating data from all randomly assigned animals who had both a baseline measurement and at least one postbaseline observation after receiving the study medication or placebo. A secondary analysis included all randomly assigned animals who completed the full study treatment period. To address missing data, multiple imputation methods were performed. Descriptive statistics for continuous and categorical variables were used to summarize baseline characteristics by trial group and overall. Normality of the data was assessed using the Shapiro–Wilk test. Treatment effect differences in the primary outcome were analyzed using a repeated measures analysis of covariance (ANCOVA) model, with the change from baseline to 3 months in the Helsinki score at each scheduled postbaseline point as the dependent variable. To eliminate the influence of baseline differences between the groups, baseline scores (as well as the diagnosis of leishmaniasis) were included as covariates in the multivariate analysis. The ANCOVA model was employed to estimate the mean differences in change from baseline to month three between each cannabis group and the placebo group, along with corresponding confidence intervals and two-sided p-values. A hierarchical sequential testing procedure, beginning with the Helsinki score and followed by secondary outcomes, was applied using a Bonferroni *post hoc* test to control the type I error rate. Secondary efficacy outcomes included the change from baseline in CBPI and VCA scores, as well as safety assessments, evaluated through similar repeated measures ANCOVA models. Point estimates and standard errors are presented for longitudinal clinical outcomes. Comparisons of adverse event frequencies between groups were performed using Fisher’s exact test followed by Benjamini–Hochberg correction (FDR-BH). Correlations between clinical assessments and owner-reported outcomes were explored using Kendall’s tau-b (τb) correlation test. An alpha level of 0.05 (two-sided) was used for all pairwise tests of treatment effects. All statistical analyses were conducted using SPSS statistical software (SPSS Statistics 22.0, SPSS Inc., Chicago, Illinois, USA). Graphs were generated with GraphPad Prism 8.0 software (GraphPad Software, Boston, MA, USA). Patients were considered to have completed the trial only after receiving treatment for the full 3-month period. The selection of raters who met the training requirements to administer the instruments on site was the responsibility of the primary investigator. Group allocations were concealed from the raters. Safety analyses included all patients who had received at least one dose of either cannabis or placebo. When participants withdrew prematurely, assessments of efficacy or safety may have been conducted during visits for which data collection had not been scheduled.

## 3 Results

**FIGURE 2:**
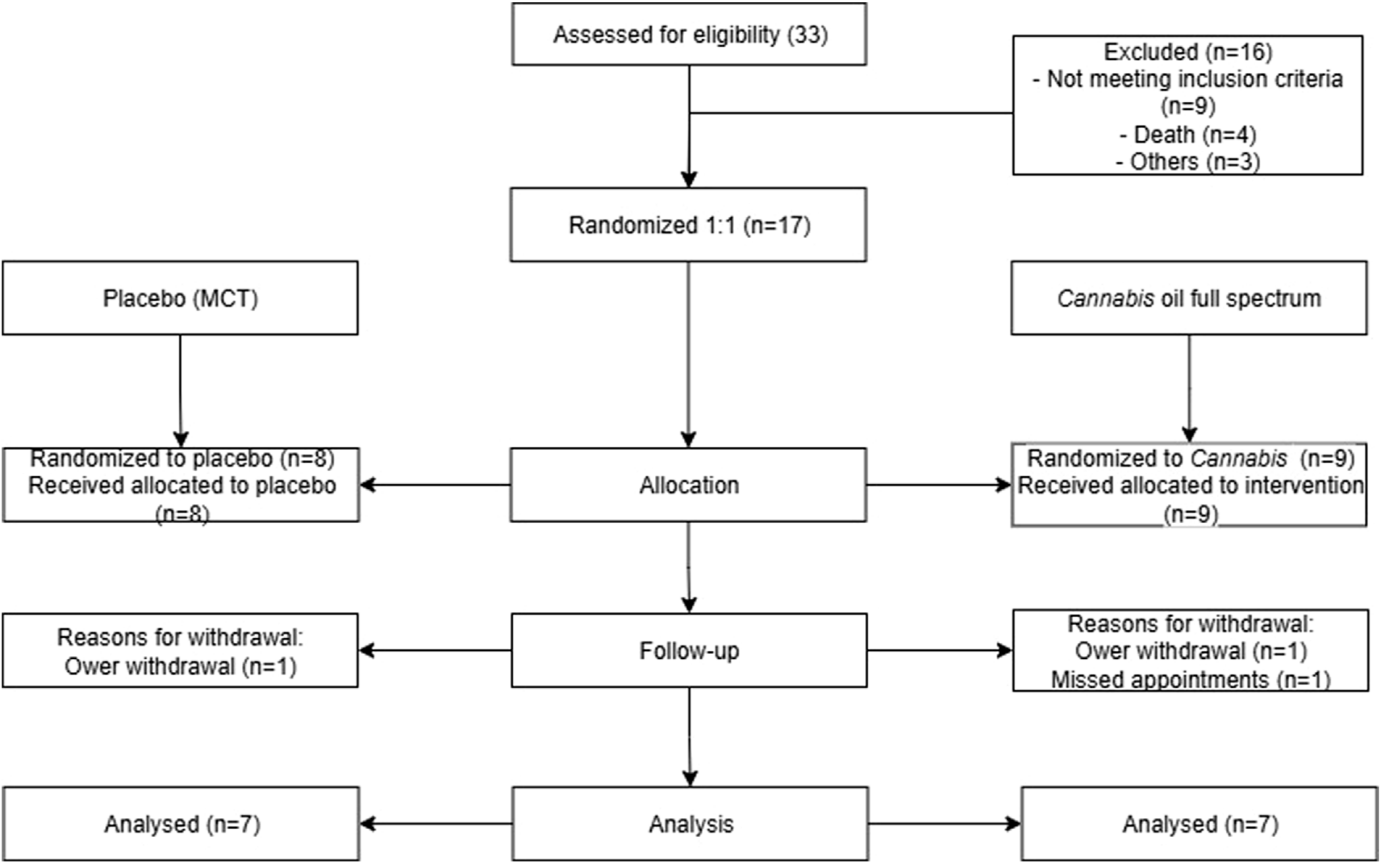
Flowchart of patient triage. This image summarizes the animal selection and study methodology over 90 days, representing the excluded animals and treatment groups.

The study flowchart is presented in Figure 2, including the number of dogs enrolled, evaluated, and included in the analysis. Dogs that met the inclusion criteria were randomly assigned to either the cannabis group (9 participants) or the placebo group (8 participants). Seven patients from each group completed the trial. Age ranged from 2 to 14 years (median 10 years), and weight ranged from 5 to 52.7 kg (median 11.6 kg). There was no significant difference between the cannabis and placebo groups when body weight and age were compared (Supplementary Figures S1, S2). A total of six dogs tested positive for leishmaniasis infection. The characteristics of the participants are detailed in Table 1.

**TABLE 1:** Characteristics of dogs enrolled in study investigating the effects of oil cannabis on osteoarthritis.

Group	Breed	Weight (kg)	Age (years)	Sex	Radiographic OA localization	CVL
Placebo	Border collie	30,35	12	F	Bilateral coxofemoral osteoarthritis	-
Placebo	Brazilian Mastiff	52,70	2	M	Bilateral coxofemoral osteoarthritis	-
Placebo	Mix breed	7,12	4	M	Bilateral coxofemoral OA, Bilateral knee OA	-
Placebo	Lhasa Apso	11,3	10	F	Bilateral coxofemoral OA	-
Placebo	Dachshund	7,4	10	F	Bilateral coxofemoral OA	+
Placebo	Lhasa Apso	8,15	9	M	Left coxofemoral OA	-
Placebo	Dachshund	9,75	13	F	Bilateral coxofemoral OA	+
Cannabis oil	German shepherd	41,5	10	F	Hind limbs coxofemoral OA	-
Cannabis oil	Mix breed	5	3	F	Bilateral coxofemoral OA	+
Cannabis oil	German Shepherd	28,4	2	F	Hind limbs coxofemoral OA	-
Cannabis oil	Mix breed	6,8	10	F	Left coxofemoral OA	+
Cannabis oil	Pug	13,7	5	M	Left coxofemoral OA	+
Cannabis oil	Mix breed	22,12	14	F	Left coxofemoral OA	-
Cannabis oil	Chow-Chow	18,2	10	F	Bilateral coxofemoral OA	-
Cannabis oil	Mix breed	5	7	F	Left coxofemoral OA and bilateral knee OA	-
Cannabis oil	Shih Tzu	11,6	12	M	Bilateral coxofemoral OA	-

F, female; M, male; CVL, canine visceral leishmaniasis.

### 3.1 Clinical assessment

#### 3.1.1 Helsinki chronic pain index

**FIGURE 3:**
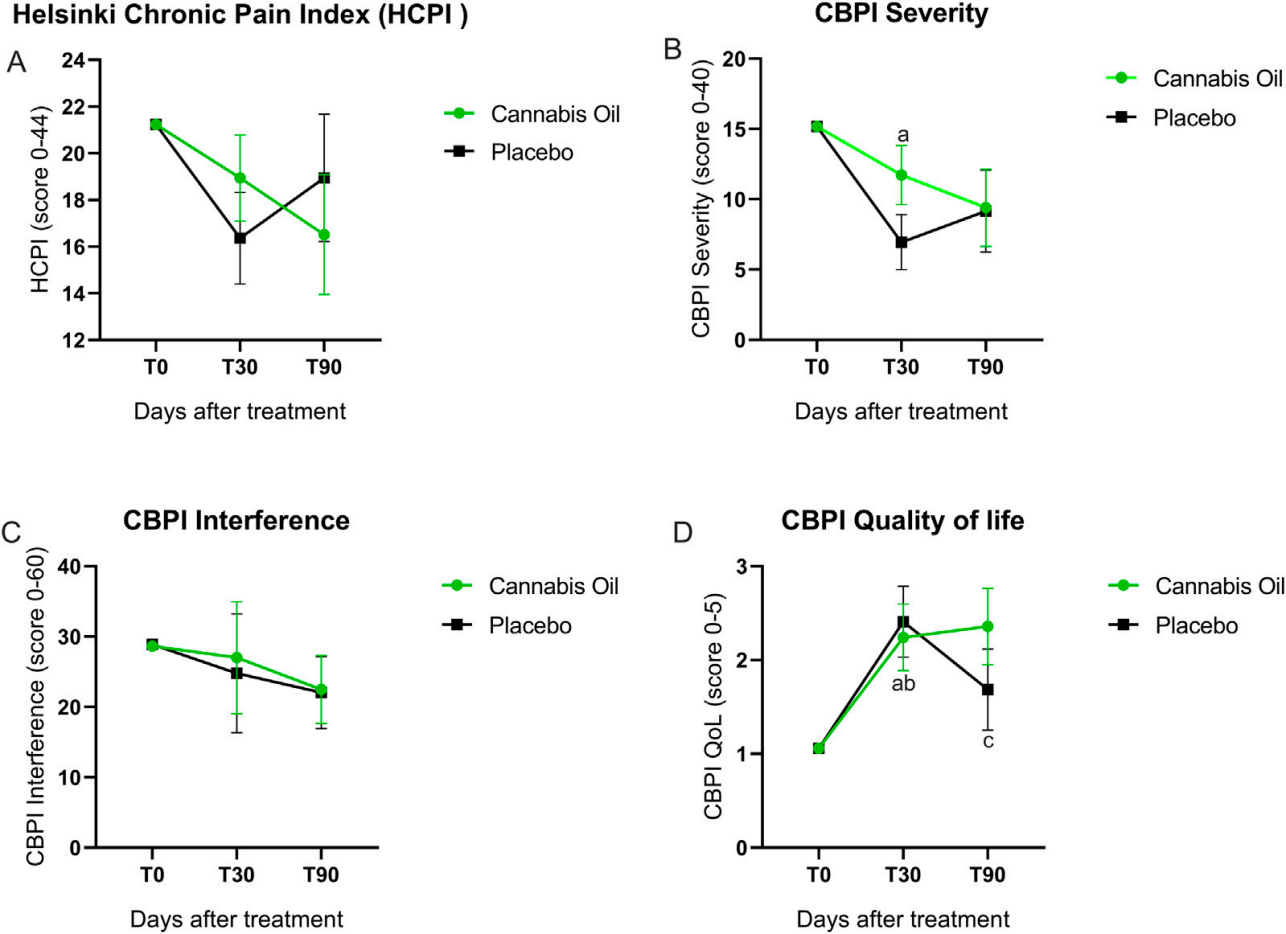
Graphical representation of HCPI and CBPI outcomes.

The Helsinki Chronic Pain Index was completed monthly by the owners. As shown in Figure 3A and Tables 2–4, no statistically significant differences were observed between the cannabis and placebo groups [F (1,14) = 0.001, p = 0.981, η^2^ = 0.000]. Similarly, the longitudinal analysis did not detect significant differences over time [F (2,28) = 1.238, p = 0.305, η^2^ = 0.081]. It is noteworthy that the mean difference in scores between the placebo and cannabis groups at day 90 was −2.4 (95% CI: −10.5 to 5.6).

**TABLE 2:** Primary and secondary clinical outcomes for the modified intention to treat population from baseline to month 3.

Outcome	Placebo			Cannabis oil		
	T0	T30	T90	T0	T30	T90
	M CI	M CI	M CI	M CI	M CI	M CI
Helsinki Chronic Pain Index (HCPI)	21,235 (21,235–21,235)	16,368 (12,166–20,571)	18,950 (13,103–24,797)	21,235 (21,235–21,235)	18,943 (14,985–22,90)	16,520 (11,014–22,02)
CBPI Severity	15,176 (15,176–15,176)	6,947 (2,160–11,735)^a^	9,156 (2,896–15,416)	15,176 (15,176–15,176)	11,727 (7,225–16,228)	9,403 (2,896–15,416)
CBPI Interference	28,647 (28,647–28,647)	24,788 (6,682–42,894)	22,037 (11,068–33,006)	28,647 (28,647–28,647)	27,007 (9,940–44,075)	22,487 (12,147–32,827)
Quality of life	1,059 (1,059–1,059)	2,408 (1,600–3,217)^a^	1,686 (0.761–2,611)	1,059 (1,059–1,059)	2,240 (1,478–3,002)^b^ ^,^ ^c^	2,358 (1,486–3,230)
Joint mobility	2,059 (2,059–2,059)	1,914 (1,249–2,579)	2,039 (1,359–2,719)	2,059 (2,059–2,059)	1,910 (1,283–2,537)	1,965 (1,325–2,606)
Lameness	2,059 (2,059–2,059)	2,263 (1,663–2,864)	2,329 (1,627–3,030)	2,059 (2,059–2,059)	1,607 (1,043–2,171)	1,549 (0.891–2,207)
Pain on palpation	1,471 (1,471–1,471)	1,042 (0.787–1,297)^a^	1,214 (0.923–1,505)	1,471 (1,471–1,471)	1,185 (0.945–1,424)	1,143 (0.870–1,416)
Weight-bearing	1,824 (1,824–1,824)	1,662 (0.918–2,405)	1,925 (1,298–2,552)	1,824 (1,824–1,824)	1,412 (0.717–2,107)	1,432 (0.846–2,018)
Overall score clinical condition	44,912 (4,912–4,912)	22,469 (0.922–4,107)	3,915 (2,235–5,595)	4,912 (4,912–4,912)	3,527 (2,071–4,984)	2,355 (0.774–3,937)

Data presented at mean (M) more 95% confidence intervals (CI). Significant differences are indicated with symbols. Exact p-values are reported in Table 4. *Represents significant differences (p < 0.05) cannabis oil versus placebo. Lameness was scored as follows: 1 = walks normally, 2 = slightly lame when walking, 3 = moderately lame when walking, 4 = severely lame when walking, 5 = reluctant to rise and will not walk more than five paces. Joint mobility was scored as follows: 1 = full range motion, 2 = Mild limitation in range of motion, no crepitus, 3 = Mild limitation in range of motion, with crepitus, 4 = Moderate limitation in range of motion, +- crepitus, 5 = Severe limitation in range of motion + - crepitus. Pain on palpation was scored as follows: 1 = none, 2 = mild signs, dog turns head in recognition, 3 = moderate signs, dog pulls limb away, 4 = severe signs, dog vocalizes or becomes aggressive, 5 = dog will not allow palpation. Weight-bearing was scored as follows: 1 = equal on all limbs standing and walking, 2 = normal standing, favors affected limb when walking, 3 = partial weight-bearing standing and walking, 4 = partial weight-bearing standing, non-weight-bearing walking, 5 = non-weight-bearing standing and walking. Overall score of clinical condition was scored as follows: 1 = not affected, 2 = milddly affected, 3 = moderately affected, 4 = severely affected, 5 = very severely affected. Helsinki Chronic Pain Index (HCPI) was scored as follows: 0–11 = no of chronic pain, 12–44 = chronic pain. *Represents significant differences (p < 0.05) cannabis oil versus placebo.

^a^ Represents significant differences (p < 0,05) between times 0 and 30 of the placebo group.

^b^ Represents significant differences (p < 0,05) between times 0 and 30 of the Cannabis oil group.

^c^ Represents significant differences (p < 0,05) between times 0 and 90 of the Cannabis oil group.

#### 3.1.2 CBPI questionnaire

##### 3.1.2.1 Pain severity

Pain severity outcomes are illustrated in Figure 3B and detailed in Tables 2–4. The analysis comparing treatment groups did not show any statistically significant differences after 90 days of treatment [F (1,14) = 0.725, p = 0.409, η^2^ = 0.049]. Likewise, the time effect analysis did not reach statistical significance [F (2,28) = 1.061, p = 0.360, η^2^ = 0.070] (Table 3). Although the overall ANCOVA model failed to achieve significance, *post hoc* comparisons using the Bonferroni test were conducted as an exploratory analysis to detect pointwise differences. A significant difference was observed in the placebo group between baseline and 30 days (p = 0.007) (Figure 3B).

##### 3.1.2.2 Pain interference

The pain interference results are presented in Figure 3C and Tables 2–4. The treatment effect analysis did not reveal significant differences between the groups after 90 days of treatment [F (1,14) = 0.036, p = 0.852, η^2^ = 0.003]. Equally, the time effect analysis showed no significant changes over time [F (2,28) = 0.966, p = 0.393, η^2^ = 0.065] (Table 3).

**TABLE 3:** ANCOVA model for primary and secondary clinical outcomes: treatment and time effects analyses.

Outcome	Treatment effect			Time effect		
	F (DF)	p-value	η^2^	F (DF)	p-value	η^2^
Helsinki Chronic Pain Index (HCPI)	F (1,14) = 0.001	0.981	0.000	F (2,28) = 1.238	0.305	0.081
CBPI Severity	F (1,14) = 0.725	0.409	0.049	F (2,28) = 1.061	0.360	0.070
CBPI Interference	F (1,14) = 0.036	0.852	0.003	F (2,28) = 0.966	0.393	0.065
Quality of life	F (1,14) = 0.272	0.610	0.019	F (2,28) = 1.280	0.294	0.084
Joint mobility	F (1,14) = 0.008	0.928	0.001	F (2,28) = 0.026	0.974	0.002
Lameness	F (1,14) = 3.417	0.086	0.196	F (2,28) = 1.983	0.157	0.124
Pain on palpation	F (1,14) = 0.065	0.803	0.005	F (2,28) = 0.583	0.565	0.040
Weight-bearing	F (1,14) = 0.701	0.416	0.048	F (2,28) = 0.692	0.509	0.047
Overall score clinical condition	F (1,14) = 0.008	0.928	0.001	F (2,28) = 0.028	0.972	0.002

Results from the ANCOVA model assessing primary and secondary clinical outcomes. Treatment effect analyses compare differences across intervention groups, while time effect analyses assess changes over time. Effect sizes are reported as η^2^ (eta squared).

##### 3.1.2.3 Correlation analyses

Correlation analyses at day 90 in the cannabis-treated group showed significant associations between owner-reported and clinician-assessed outcomes. A strong positive correlation was observed between HCPI and the CBPI total score (τb = 0.771, p = 0.004). Pain on palpation assessed by the veterinarian correlated with the CBPI pain intensity subscore (τb = 0.642, p = 0.039). CBPI pain intensity was also correlated with the CBPI interference subscore (τb = 0.580, p = 0.034). No other clinical instrument yielded significant correlations (data not shown). These results indicate consistency between caregiver questionnaires and clinical examination, supporting their complementary use in assessing pain in dogs with osteoarthritis.

##### 3.1.2.4 Quality of life

No statistically significant differences in quality of life were identified between the treatment groups after 90 days [F (1,14) = 0.272, p = 0.610, η^2^ = 0.019]. In addition, the longitudinal analysis did not demonstrate significant variation across time points [F (2,28) = 1.280, p = 0.294, η^2^ = 0.084] (Tables 2, 3). In the exploratory *post hoc* analysis (Table 4), a statistically significant improvement was observed in the placebo group between baseline and 30 days (p = 0.009). Additionally, in the cannabis group, significant differences were found between baseline and 30 days (p = 0.015), as well as between baseline and 90 days (p = 0.019) (Figure 3D).

**TABLE 4:** Bonferroni *post hoc* test exact p-values for primary and secondary clinical outcomes.

Outcome	Treatment effect Placebo vs. cannabis	Time effect Placebo group			Time effect Cannabis group		
	p-value	p-value T0 vs. T30	p-value T0 vs. T90	p-value T30 vs. T90	p-value T0 vs. T30	p-value T0 vs. T90	p-value T30 vs. T90
Helsinki Chronic Pain Index (HCPI)	0.531	0.079	1.000	0.734	0.703	0.263	0.739
CBPI Severity	0.953	0.007	0.175	1.000	0.368	0.162	1.000
CBPI Interference	0.950	1.000	0.651	1.000	1.000	0.666	1.000
Quality of life	0.276	0.009	0.504	0.283	0.015	0.019	1.000
Joint mobility	0.868	1.000	1.000	0.684	1.000	1.000	1.000
Lameness	0.117	1.000	1.000	1.000	0.323	0.357	1.000
Pain on palpation	0.719	0.009	0.240	0.935	0.068	0.066	1.000
Weight-bearing	0.267	1.000	1.000	0.652	0.674	0.522	1.000
Overall score clinical condition	0.966	0.307	0.725	0.460	0.891	1.000	0.891

Pairwise comparisons conducted using the Bonferroni correction. Mean differences and exact p-values are reported for each time point and group comparison. p-values <0.05 indicate significant differences.

Animals treated with the cannabis extract exhibited an improvement in quality of life. The proportion of dogs classified as having a “very good” quality of life increased by approximately 15%, while those rated as “excellent” increased by 30%. Notably, at the end of the trial, none of the cannabis-treated dogs were classified as having a poor quality of life. In contrast, the initial values in the placebo group remained unchanged throughout the study. These results suggest a significant enhancement in the quality of life of the dogs treated with the cannabis extract.

#### 3.1.3 Veterinary clinical assessments (VCA)

**FIGURE 4:**
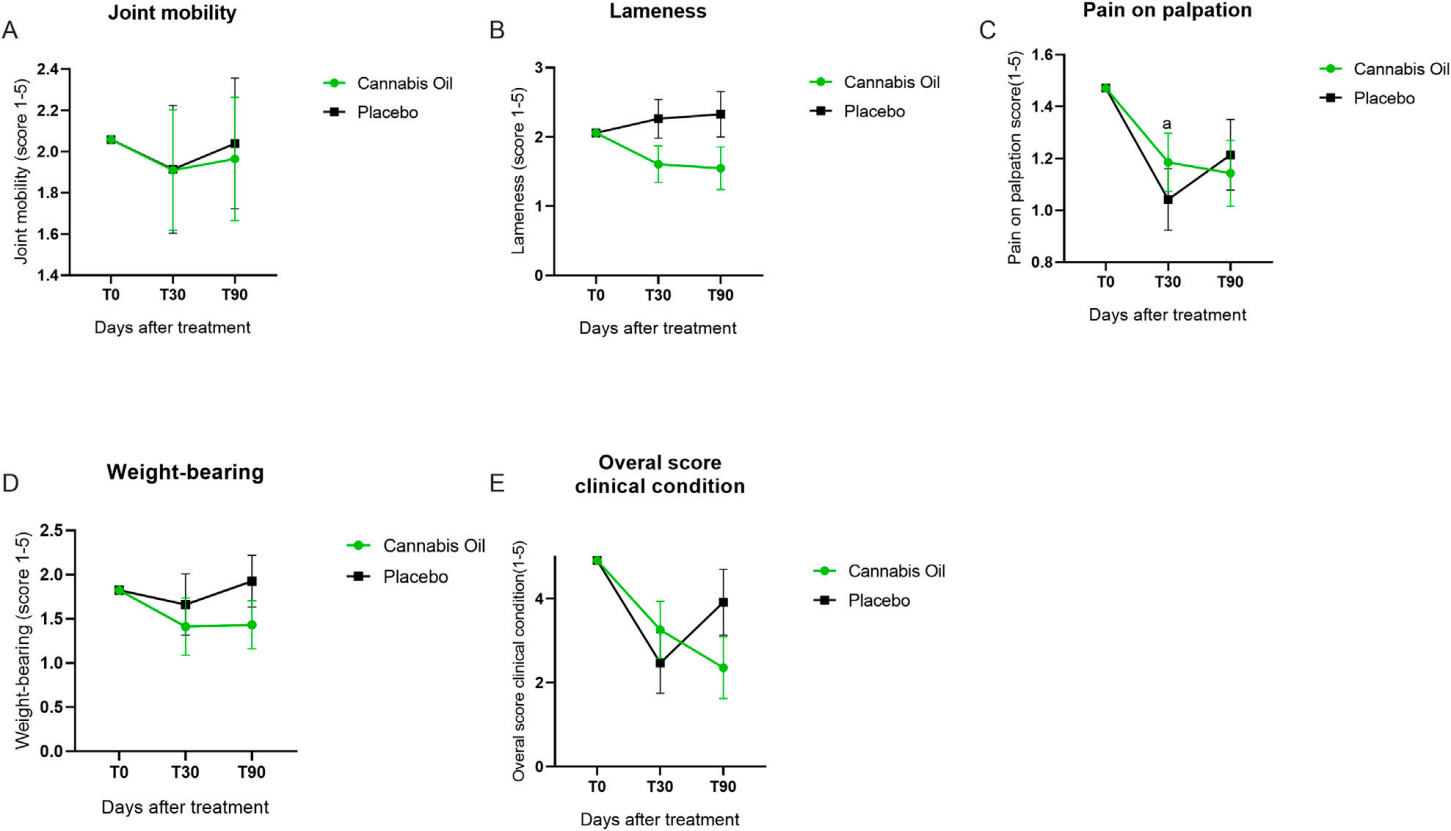
Graphical summary of veterinary clinical assessment outcomes.

The veterinary clinical assessment (VCA) was conducted to evaluate joint function and pain-related parameters. No statistically significant differences were detected for joint mobility [F (1,14) = 0.008, p = 0.928, η^2^ = 0.001] (Figure 4A), lameness [F (1,14) = 3.417, p = 0.086, η^2^ = 0.196] (Figure 4B), pain on palpation [F (1,14) = 0.065, p = 0.803, η^2^ = 0.005] (Figure 4C), or weight-bearing [F (1,14) = 0.701, p = 0.416, η^2^ = 0.048] (Figure 4D). In the Bonferroni *post hoc* analysis, however, a significant intragroup difference was observed for pain on palpation in the placebo group between baseline and 30 days (p = 0.009) (Figure 4C). The overall clinical condition, as assessed by the VCA, also showed no statistically significant effects in either treatment or time effects analyses. The comparison between treatment groups revealed no differences after 90 days [F (1,14) = 0.008, p = 0.928, η^2^ = 0.001], and longitudinal evaluation across time points did not indicate any significant change [F (2,28) = 0.028, p = 0.972, η^2^ = 0.002] (Figure 4E; Tables 2, 3).

### 3.2 Safety assessments

#### 3.2.1 Adverse effects assessment

Throughout the study, owners maintained a record of any adverse events experienced by the patients. During individual meetings with the owners, a notebook containing information on the administration of the medication was provided, along with the contact details of the veterinarian in charge for reporting any serious adverse events. Owners were instructed to be particularly observant during the first hour after administration. The notebook included a list of possible adverse events that could occur. At each consultation, the notebook was reviewed, and the reported reactions were duly cataloged.

A total of 64 adverse events were reported, with 25 occurring in the placebo group and the remaining 39 in the cannabis group. Diarrhea was the most frequently reported event, with 12 cases in the cannabis group compared to 22 in the placebo group. Notably, seven events of anxiety (p = 0.005) and six events of hyperphagia (p = 0.023) were reported in the cannabis-treated group (Supplementary Table S1). These were the only adverse events that reached statistical significance. Importantly, all adverse events were classified as mild, required no veterinary intervention, and resolved spontaneously within 24 h. These findings further support the safety of the cannabis-based formulation, as no severe or medically significant adverse effects were observed in the dogs with osteoarthritis evaluated in this study.

#### 3.2.2 Biochemical safety assessment

**FIGURE 5:**
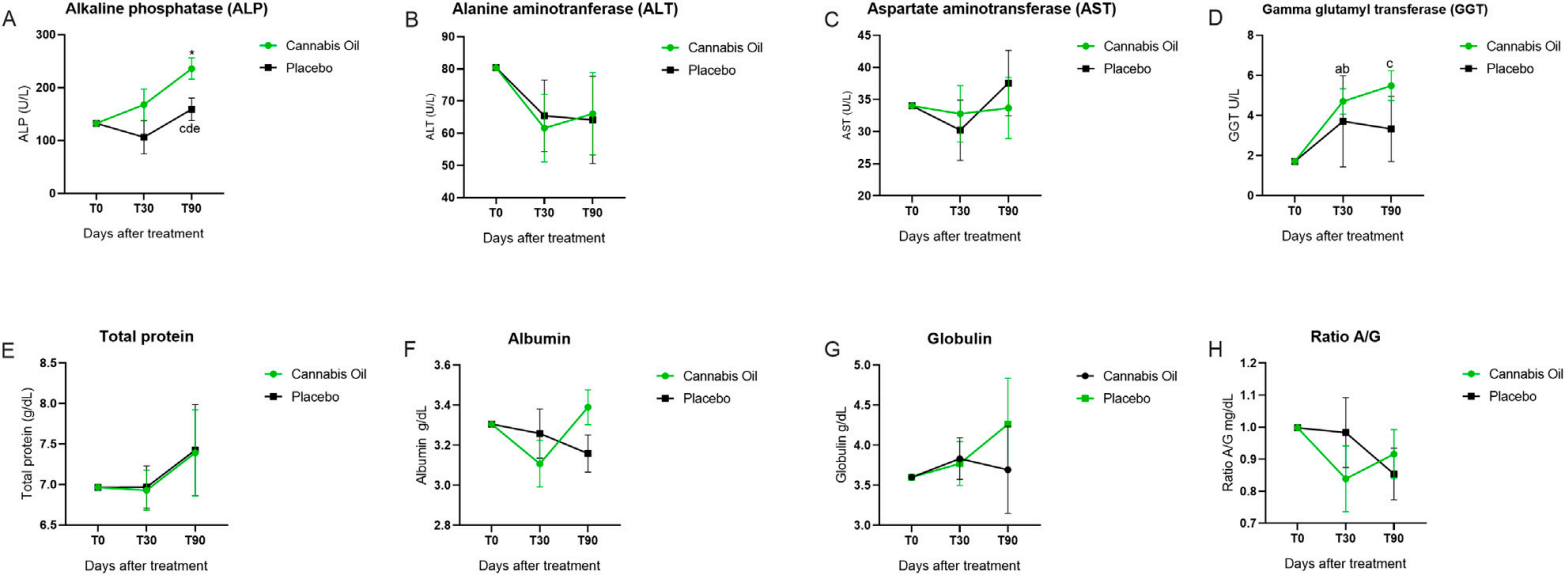
Biochemical marker levels during the 90-day treatment period. Part I. *p < 0.05 for significant differences between cannabis oil and placebo groups. **(A)** p < 0.05 for significant differences between time points 0 and 30 within the placebo group. **(B)** p < 0.05 for significant differences between time points 0 and 30 within the cannabis oil group. **(C)** p < 0.05 for significant differences between time points 0 and 90 within the cannabis oil group. **(D)** p < 0.05 for significant differences between time points 30 and 90 within the cannabis oil group. **(E)** p < 0.05 for significant differences between time points 30 and 90 within the placebo group.

**FIGURE 6:**
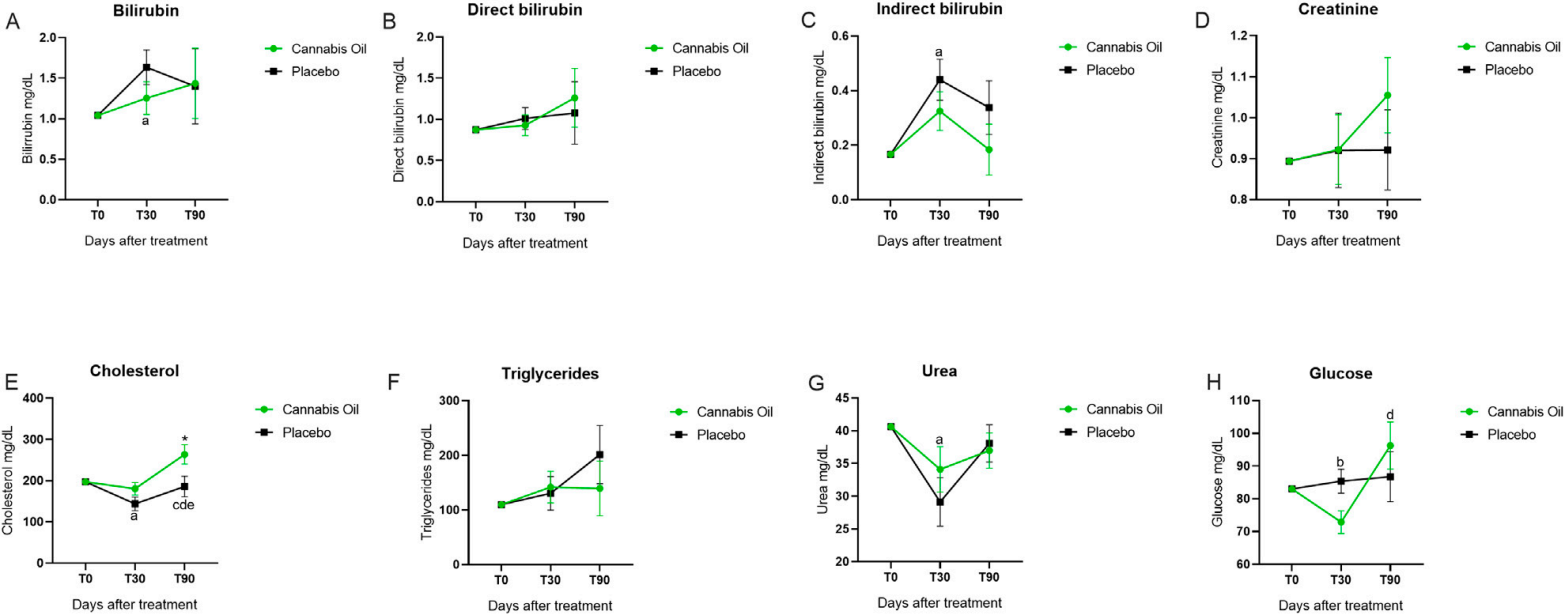
Biochemical marker levels during the 90-day treatment period. Part II. *p < 0.05 for significant differences between cannabis oil and placebo groups. **(A)** p < 0.05 for significant differences between time points 0 and 30 with in the placebo group. **(B)** p < 0.05 for significant differences between time points 0 and 30 with in the cannabis oil group. **(C)** p < 0.05 for significant differences between time points 0 and 90 with in the cannabis oil group. **(D)** p < 0.05 for Q27 significant differences between time points 30 and 90 with in the cannabis oil group. **(E)** p < 0.05 for significant differences between time points 30 and 90 with in the placebo group.

Serum biochemical markers, including AST, ALT, ALP, GGT, total bilirubin, direct bilirubin, indirect bilirubin, urea, creatinine, cholesterol, and glucose, were monitored throughout the study (Figures 5A–H; Figures 6A–H; Table 5).

**TABLE 5:** Summary of the serum biochemical markers levels from baseline to month 3.

Biomarker	Placebo				*Cannabis* oil		
	REF.	T0	T30	T90	T0	T30	T90
		M CI	M CI	M CI	M CI	M CI	M CI
Creatinine	0,5–1.4 mg/dL	0,89 (0,89–0,89)	0,92 (0,72–1,11)	0,92 (0,71–1,13)	0,89 (0,89–0,89)	0,92 (0,73–1,10)	1,05 (0,85–1,25)
ALT	10–88 U.I./L	80,41 (80,41–80,41)	65,43 (41,52–89,33)	64,15 (35,04–93,27)	80,41 (80,41–80,41)	61,58 (39,06–84,10)	66,08 (38,65–93,50)
AST	10–88 U.I./L	34,00 (34,00–34,00)	30,235 (20,15–40,31)	37,56 (26,63–48,49)	34,00 (34,00–34,00)	32,79 (23,36–42,22)	33,67 (23,44–43,90)
ALP	20–150 U.I./L	132,67 (132,67–132,67)	106,25 (39,23–173,26)	159,09 (113,34–204,84)^e^	132,67 (132,67–132,67)	167,83 (104,68–230,99)	235,95 (192,84–279,07)*^c^ ^,^ ^d^
GGT	1,2–8 U.I./L	1,71 (1,71–1,71)	3,70 (2,27–5,14)^a^	3,32 (1,62–5,03)	1,71 (1,71–1,71)	4,70 (3,35–6,05)^b^	5,48 (3,87–7,08)^c^
Total protein	6–8 g/dL	6,96 (6,96–6,96)	6,96 (6,40–7,52)	7,42 (6,22–8,63)	6,96 (6,96–6,96)	6,92 (6,39–7,45)	7,39 (6,25–8,53)
Albumin	2,3–3.8 g/dL	3,30 (3,30–3,30)	3,25 (2,99–3,52)	3,15 (2,96–3,35)	3,30 (3,30–3,30)	3,10 (2,85–3,35)	3,39 (3,20–3,57)
Globulin	2,5–4.5 g/dL	3,60 (3,60–360)	3,77 (3,18–4,35)	4,26 (3,02–5,50)	3,60 (3,60–360)	3,83 (3,27–4,38)	3,69 (2,52–4,86)
Ratio A/G	0,5–1.7 mg/dL	0,99 (0,99–0,99)	0,98 (0,74–1,21)	0,85 (0,68–1,02)	0,99 (0,99–0,99)	0,83 (0,61–1,05)	0,91 (0,75–1,07)
Bilirrubin	0,1–0.7 mg/dL	1,04 (1,04–1,04)	1,63 (1,17–2,09)^a^	1,40 (0,40–2,39)	1,04 (1,04–1,04)	1,25 (0,82–1,68)	1,43 (0,50–2,36)
Direct bilirubin	0,06–0.3 mg/dL	0,87 (0,87–0,87)	1,01 (0,72–1,29)	1,076 (0,26–1,88)	0,87 (0,87–0,87)	0,92 (0,65–1,19)	1,26 (0,48–2,02)
Indirect bilirubin	0,01–0.5 mg/dL	0,16 (0,16–0,16)	0,44 (0,27–0,60)^a^	0,33 (0,12–0,54)	0,16 (0,16–0,16)	0,32 (0,17–0,47)	0,18 (−0.015–0,38)
Cholesterol	135–270 mg/dL	197,23 (197,23–197,23)	143,74 (109,00–178,48)^a^	185,74 (132,24–239,25)^e^	197,23 (197,23–197,23)	180,41 (147,70–213,13)	263,52 (213,14–313,89)*^c^ ^,^ ^d^
Triglycerides	15–130 mg/dL	109,87 (109,87–109,87)	130,54 (64,45–196,63)	201,67 (87,45–315,89)	109,87 (109,87–109,87)	141,94 (79,73–204,15)	139,61 (32,09–247,13)
Urea	10–60 mg/dL	40,62 (40,62–40,62)	29,51 (21,15–37,87)^a^	38,05 (31,61–44,50)	40,62 (40,62–40,62)	34,03 (26,18–41,89)	37,01 (30,96–43,07)
Glucose	60–110 mg/dL	83,04 (83,04–83,04)	85,39 (77,51–93,28)	86,74 (70,40–103,08)	83,04 (83,04–83,04)	72,89 (65,45–80,33)^b^	96,28 (80,88–111,69)^c^

Data presented at mean (M) and confidence intervals (CI). Significant differences are indicated with symbols. Exact p-values are reported in Table 7. * Represents a significant difference (p < 0.05) between cannabis oil and placebo at time 90.

^a^ represents significant differences (p < 0,05) between times 0 and 30 of the placebo group.

^b^ represents significant differences (p < 0,05) between times 0 and 30 of the Cannabis oil group.

^c^ represents significant differences (p < 0,05) between times 0 and 90 of the Cannabis oil group.

^d^ represents significant differences (p < 0,05) between times 30 and 90 of the Cannabis oil group.

^e^ represents significant differences (p < 0,05) between times 30 and 90 of the placebo group.

For the main safety-related analytes, no treatment effects were detected for AST [F (1,14) = 0.011, p = 0.920, η^2^ = 0.001] (Figure 5C), ALT [F (1,14) = 0.003, p = 0.955, η^2^ = 0.000] (Figure 5B), urea [F (1,14) = 0.238, p = 0.633, η^2^ = 0.017] (Figure 6G), or creatinine [F (1,14) = 0.341, p = 0.569, η^2^ = 0.024] (Figure 6D) (Table 6). Analysis of time effects showed significant changes for cholesterol [F (2,28) = 4.216, p = 0.025, η^2^ = 0.231] and glucose [F (2,28) = 3.515, p = 0.043, η^2^ = 0.201]. In addition, Bonferroni post hoc tests were performed as part of the exploratory analysis (Table 7).

**TABLE 6:** ANCOVA model for serum biochemical markers: treatment and time effects analyses.

Biomarker	Treatment effect			Time effect		
	F (DF)	p-value	η^2^	F (DF)	p-value	η^2^
Creatinine	F (1,14) = 0.341	0.569	0.024	F (2,28) = 0.646	0.532	0.044
ALT	F (1,14) = 0.003	0.955	0.000	F (2,28) = 0.078	0.925	0.006
AST	F (1,14) = 0.011	0.920	0.001	F (2,28) = 0.467	0.632	0.032
ALP	F (1,14) = 3.994	0.065	0.222	F (2,28) = 2.953	0.069	0.174
GGT	F (1,14) = 4.045	0.064	0.224	F (2,28) = 1.780	0.187	0.113
Total protein	F (1,14) = 0.006	0.941	0.000	F (2,28) = 0.002	0.998	0.000
Albumin	F (1,14) = 0.124	0.730	0.009	F (2,28) = 2.488	0.101	0.151
Globulin	F (1,14) = 0.278	0.606	0.019	F (2,28) = 0.519	0.601	0.036
Ratio A/G	F (1,14) = 0.183	0.675	0.013	F (2,28) = 1.002	0.380	0.067
Bilirrubin	F (1,14) = 0.178	0.680	0.013	F (2,28) = 0.370	0.694	0.026
Direct bilirrubin	F (1,14) = 0.061	0.809	0.004	F (2,28) = 0.300	0.743	0.021
Indirect bilirrubin	F (1,14) = 2.277	0.154	0.140	F (2,28) = 0.689	0.510	0.047
Cholesterol	F (1,14) = 4.420	0.054	0.240	F (2,28) = 4.216	0.025	0.231
Triglycerides	F (1,14) = 0.203	0.659	0.014	F (2,28) = 1.004	0.379	0.067
Urea	F (1,14) = 0.238	0.633	0.017	F (2,28) = 0.790	0.464	0.053
Glucose	F (1,14) = 0.044	0.836	0.003	F (2,28) = 3.515	0.043	0.201

Results from the ANCOVA model assessing serum biochemical markers levels. Treatment effect analyses compare differences across intervention groups, while time effect analyses assess changes over time. Effect sizes are reported as η^2^ (eta squared).

**TABLE 7:** Bonferroni *post hoc* test exact p-values for serum biochemical markers.

Biomarker	Treatment effect Placebo vs. cannabis	Time effect Placebo group			Time effect Cannabis group		
	p-value	p-value T0 vs. T30	p-value T0 vs. T90	p-value T30 vs. T90	p-value T0 vs. T30	p-value T0 vs. T90	p-value T30 vs. T90
Creatinine	0.353	1.000	1.000	1.000	1.000	0.298	0.468
ALT	0.920	0.601	0.753	1.000	0.284	0.844	1.000
AST	0.608	1.000	1.000	0.265	1.000	1.000	1.000
ALP	0.020	1.000	0.707	0.037	0.757	0.000	0.005
GGT	0.074	0.030	0.185	1.000	0.001	0.001	1.000
Total protein	0.967	1.000	1.000	1.000	1.000	1.000	1.0000
Albumin	0.097	1.000	0.409	1.000	0.330	1.000	0.184
Globulin	0.486	1.000	0.811	1.000	1.000	1.000	1.000
Ratio A/G	0.586	1.000	0.291	1.000	0.432	0.904	1.000
Bilirrubin	0.957	0.042	1.000	1.000	0.907	1.000	1.000
Direct bilirrubin	0.732	0.959	1.000	1.000	1.000	0.880	0.939
Indirect bilirrubin	0.276	0.008	0.314	1.000	0.127	1.000	0.678
Cholesterol	0.041	0.016	1.000	0.047	0.866	0.041	0.000
Triglycerides	0.416	1.000	0.320	0.134	0.862	1.000	1.000
Urea	0.785	0.027	1.000	0.133	0.247	0.585	1.000
Glucose	0.377	1.000	1.000	1.000	0.033	0.259	0.004

Pairwise comparisons conducted using the Bonferroni correction. Mean differences and exact p-values are reported for each time point and group comparison. p-values <0.05 indicate significant differences.

Post hoc analysis indicated that cholesterol decreased in the placebo group from baseline to day 30 (p = 0.016) and increased from day 30 to day 90 (p = 0.047), while in the cannabis group it increased from baseline to day 90 (p = 0.041) and from day 30 to day 90 (p < 0.001) (Figure 6E). At day 90, cholesterol was also higher in the cannabis group compared with the placebo group (p = 0.041). For glucose, values decreased in the cannabis group from baseline to day 30 (p = 0.033) and increased from day 30 to day 90 (p = 0.004) (Figure 6H). Because the overall ANCOVA models were significant, these *post hoc* findings complement the global results. Despite these changes, both cholesterol and glucose remained within the reference range.

ALP values were above the reference range in both groups after trial initiation. Post hoc analyses showed an increase in the placebo group between day 30 and day 90 (p = 0.037) (Figure 5A) and in the cannabis group from baseline to day 90 (p < 0.001) and from day 30 to day 90 (p = 0.005). Total bilirubin, direct bilirubin, indirect bilirubin, and triglycerides also exceeded the reference range in both groups. Exploratory *post hoc* tests identified an increase in total bilirubin from baseline to day 90 in the placebo group (p = 0.042) (Figure 6A) and an increase in indirect bilirubin from baseline to day 30 in the placebo group (p = 0.008) (Figure 6C), while no significant comparisons were observed for direct bilirubin (Figure 6B) or triglycerides (Figure 6F). Since the overall ANCOVA time effects for these analytes were not significant, the *post hoc* results are considered non-confirmatory.

In summary, ANCOVA detected no treatment effects for the main safety markers (AST, ALT, urea, creatinine) and revealed significant time effects for cholesterol and glucose, although values remained within the reference range. Exploratory *post hoc* analyses identified differences for ALP and bilirubin, both of which exceeded reference ranges. Despite these biochemical alterations, none of the animals exhibited clinical signs of hepatic or renal dysfunction.

#### 3.2.3 Glasgow coma scale

None of the animals in the study showed any changes in the Glasgow Coma Scale. Thus, all animals in both groups maintained a score of 15 on this scale throughout the entire study period (Table 8).

**TABLE 8:** Glasgow coma scale score.

Time	Placebo group	Treated group
T 0	15	15
T 30	15	15
T 90	15	15

The data represent the values obtained from the veterinary neurological assessment conducted during the study, using the Glasgow Coma Scale. Score 0–15, with a higher score indicating a better neurological assessment.

## 4 Discussion

Here we describe a randomized, double-blind, placebo-controlled study evaluating the safety and efficacy of a full-spectrum cannabis oil containing primarily CBD and THC in dogs with osteoarthritis. We demonstrated that a 90-day daily treatment was clinically and biochemically safe for the animals. Furthermore, although the treatment was not statistically effective in reducing pain in these animals, the numerical reduction in pain levels in the cannabis group suggests that cannabis has the potential to alleviate pain and improve quality of life in these patients, warranting further studies with larger sample sizes and additional methodologies.

In our study, dogs treated with cannabis exhibited a reduction of 2.4 points in pain relief as measured by the Helsinki Chronic Pain Index (HCPI) (Hielm-Björkman et al., 2009) compared to placebo, although the results were not statistically significant. In the treatment effect analysis, the HCPI scores for joint mobility, lameness, and weight-bearing capacity showed no significant changes over the study period. In a similar study, Verrico and colleagues (2020) reported significant pain reduction via the HCPI only at higher CBD doses (>20 mg/day), but not at lower doses (20 mg/day). Some studies (e.g., Brioschi et al., 2020) suggest that owner-reported assessments (HCPI) may detect subtler clinical improvements compared to veterinarian evaluations, potentially due to the owners’ continuous observation of behavioral changes in home environments, such as the “Hawthorne effect” (McCambridge et al., 2014). This sensitivity difference could explain the progressive (albeit non-significant) trends observed in our cannabis group’s HCPI data despite negative categorical results. Additionally, significant correlations at day 90 indicate alignment between owner-reported outcomes (HCPI, CBPI) and veterinary assessment of pain, supporting the validity of combining these instruments in the evaluation of osteoarthritis in dogs.

When we used the CBPI method to evaluate pain severity, pain interference, and quality of life, we also found no changes in any of these symptoms. The CBPI is an owner-reported assessment that evaluates behavioral and locomotor parameters to estimate pain levels. A previous study by another research group assessed the effects of isolated CBD in dogs with osteoarthritis (OA) and found that CBD (4 mg/kg per day) did not significantly reduce pain scores or improve quality of life (Brioschi et al., 2020). Similarly, Mejia et al. (2021) evaluated dogs treated with a full-spectrum CBD oil (2.5 mg/kg per day over 6 weeks) using the Liverpool Osteoarthritis in Dogs pain scale and observed no statistically significant difference compared to placebo. It is possible that a larger sample size might have yielded significant results, as also suggested by Brioschi et al. (2020). Additionally, Verrico and colleagues (2020) reported that dogs receiving 20 mg/day of CBD showed no significant improvement in pain relief based on owner assessments using the HCPI. On the other hand, several studies administering different cannabinoids in rats or mice subjected to MIA-induced osteoarthritis models have demonstrated that these compounds significantly reduced pain and inflammation levels (O’Brien and McDougall, 2018). Specifically, CBD (Philpott et al., 2017), CB1 agonists (Schuelert and McDougall, 2008), CB2 agonists (Yao et al., 2008; Schuelert et al., 2010), as well as FAAH inhibitors (Ahn et al., 2011; McDougall et al., 2017), have been shown to reduce pain, allodynia, or nociceptive fiber firing in animal models of osteoarthritis. These findings from animal and human studies, combined with the well-documented efficacy of cannabinoids in alleviating various types of pain (Nutt et al., 2022; Silva-Cardoso and Leite-Panissi, 2023; Cásedas et al., 2024) and inflammation (Henshaw et al., 2021), lead us to hypothesize that cannabinoids may be effective in managing osteoarthritic pain. However, neither this study nor similar canine trials have yet identified the optimal formulation to conclusively demonstrate this effect.

When we analyze the quality of life (QoL) of dogs, which is closely linked to mobility and the ability to perform daily activities, we hypothesize that this outcome may be attributed to insufficient pain relief. Brioschi et al. (2020) similarly reported a numerical but non-significant improvement in QoL among osteoarthritic dogs, a finding consistent with our results. Further supporting this observation, a study by Gamble and collaborators (2018) evaluating cannabis treatment in dogs with OA showed that this therapy was able to increase comfort and activity in the home environment with 2 mg/kg of CBD extract administered twice daily over a 4-week period.

Regarding safety, the cannabis-based treatment demonstrated a favorable profile overall. However, two adverse effects, anxiety and hyperphagia, were significantly more frequent in the cannabis group compared to placebo. We attribute these effects to the substantial THC content in our formulation, as this cannabinoid is well documented to induce both anxiety and increased appetite (Crippa et al., 2009; Di Marzo, 2020). However, these effects were transient, observed only during the first 8 days following treatment initiation. Importantly, our biochemical analyses revealed no significant differences between the cannabis and placebo groups, further reinforcing the safety profile of this full-spectrum cannabis extract throughout the 90-day administration period.

Notably, we observed an increase in alkaline phosphatase (ALP) levels, a phenomenon previously documented in other studies following 30 days of cannabidiol (CBD) treatment (Gamble et al., 2018; Vaughn et al., 2021; Bradley et al., 2022). In our study, both the placebo and cannabis groups exhibited ALP levels above the reference range after trial initiation; these differences appeared in *post hoc* analyses but were not supported by the primary model. This elevation may relate to the use of medium chain triglycerides (MCT) oil as the vehicle, since ALP also increased in the placebo group. Previous studies and our data did not report clinically relevant adverse effects from elevated ALP. A plausible explanation is an adaptive physiological response, as compounds such as CBD can modulate cytochrome P450 enzymes (Gamble et al., 2018; Deabold et al., 2019; Mejia et al., 2021; Bookout et al., 2024; Alvarenga et al., 2024), leading to transient fluctuations in biochemical markers, including ALP (Qian et al., 2019).

Furthermore, the primary ANCOVA model indicated a time effect on cholesterol and glucose in the cannabis group; however, these were mild fluctuations that remained within the reference range, with no observable impact on clinical assessments. These observations are consistent with established pharmacological principles, where such biomarker variations typically represent physiological adaptation rather than pathological significance.

This study was conducted in a leishmaniasis-endemic region, with approximately 35% of animals in each group testing positive. Leishmaniasis, a systemic parasitic infection, is known to impair liver function and elevate liver biomarkers (Solano-Gallego et al., 2011; Lima et al., 2019). Therefore, we believe that leishmaniasis infection in many patients is responsible for the elevated bilirubin levels above the reference range observed in both groups from baseline, although the primary ANCOVA model did not reveal significant differences. Affected animals were also treated with allopurinol, a first-line therapy metabolized by CYP450 (Xu et al., 2013), which may have influenced biomarker profiles. Thus, both leishmaniasis and allopurinol use likely confounded the biochemical analysis, but since no significant alterations were observed, preexisting infection did not appear to increase susceptibility to cannabis oil exposure.

Our study advances prior research by utilizing a CBD-rich oil containing significant concentrations of additional cannabinoids, including THC, CBC, CBGA, and CBG. A key distinguishing feature of our investigation is its extended 90-day observation period, compared to the shorter 45-day and 30-day follow-ups (Verrico et al., 2020; Gamble et al., 2018). The observed analgesic effects of cannabis in several studies involve multiple mechanisms, primarily mediated through activation of both peripheral (joint-localized) and central CB1 and CB2 receptors (Valastro et al., 2017; O’Brien and McDougall, 2018). As these receptors are coupled to inhibitory G-proteins, their activation may suppress pain signaling through both peripheral/spinal and supraspinal pathways, with CB1 playing a particularly prominent role due to its expression in nociceptive nerve terminals and supraspinal pain-processing regions (Lu and Mackie 2021). Notably, while CBD does not directly act as a CB1 agonist, it exerts indirect effects on this pathway through fatty acid amide hydrolase (FAAH) inhibition (De Petrocellis et al., 2011). By reducing anandamide degradation, CBD potentiates endocannabinoid signaling via CB1 receptors, which likely contributes to its analgesic properties to some degree.

Furthermore, the activation of CB1 receptors, albeit to a lesser extent, by other cannabinoids such as CBG, CBGA, and CBC (Maione et al., 2011; Zagzoog et al., 2024) may contribute to reduced peripheral nociceptive signaling, thereby enhancing the global analgesic effect of the cannabis oil (Maione et al., 2011; De Moulin et al., 2014; Henderson-Redmond et al., 2021). Additionally, the anti-inflammatory properties mediated through multiple pathways likely play a significant role: (1) CB2 receptor activation by CBG, CBC, and THC; and (2) CBD effects mediated by mechanisms independent of cannabinoid receptors (Donvito et al., 2018). These combined actions may effectively reduce nociceptive sensitization and potentiate the overall analgesic response. We hypothesized that the observed pain reduction in our study resulted from a synergistic interaction of several factors: (1) the relatively high CBD dosage; (2) complementary effects of THC and other phytocannabinoids present in significant concentrations (particularly CBG, CBGA, and CBC); and (3) the potential contribution of minor cannabinoids and terpenoids characteristic of the full-spectrum oil formulation. However, we did not find these results. We consider that our chosen study design could have been effective if we had a larger sample size and a longer follow-up.

Our study has some important limitations. First, our sample size was small; future investigations with larger sample sizes are needed to confirm these findings with greater statistical power. Additionally, pain assessment in this study relied on owner-reported outcomes, which are inherently subjective. A critical consideration in such clinical trials is the Hawthorne effect (Hercock et al., 2009; Conzemius and Evans, 2012), wherein owners’ awareness of the treatment may unintentionally bias their observations, potentially leading to an overestimation of therapeutic efficacy. In our study, a Hawthorne effect may have influenced both the Canine Brief Pain Inventory (CBPI) and Helsinki Index scores at the 30-day follow-up. Furthermore, although the cannabis extract used in this study had its main constituents quantified, it is not a specific product available on the market for future studies with the exact same formulation. The same applies to the vehicle used to prepare the oil.

These observations emphasize the challenges of short-term evaluations and highlight the need to account for owner-related bias in veterinary clinical trials. Thus, longer-duration studies with blinded assessments are essential to minimize false-positive or false-negative results attributable to placebo effects.

Nevertheless, the *post hoc* results reported here, even when adjusted for multiplicity, should be regarded as hypothesis-generating. Although they may indicate pointwise differences that warrant further investigation, in some cases such findings were not consistently supported by the primary ANCOVA model. Chen et al. (2018), in a simulation study, examined the performance of the omnibus test (i.e., F-statistic) and argued that it is important to perform pairwise comparisons regardless of the significance of the F test. Even so, it is strongly recommended that these findings be interpreted with caution, as exploratory rather than confirmatory evidence, and they cannot be considered proof of efficacy.

In summary, our study demonstrated that a CBD-rich full-spectrum cannabis oil containing significant amounts of THC, CBG, CBGA, and CBC was clinically and biochemically safe for dogs of various breeds and sizes during 90 days of daily administration. However, we failed to establish significant efficacy in either osteoarthritis pain reduction or quality of life improvement. These findings suggest the need for larger-scale canine studies to properly evaluate the clinical potential of full-spectrum cannabis oil for osteoarthritis management in dogs. While our current results did not demonstrate statistically significant effects, they indicate a potential therapeutic benefit that warrants further investigation in expanded clinical trials.

## Data Availability

The raw data supporting the conclusions of this article will be made available by the authors, without undue reservation.

## Supplementary material

SUPPLEMENTARY FIGURE S1 Animal body weight at baseline and throughout the 90-day treatment period.

SUPPLEMENTARY FIGURE S2 Animal age at study baseline.
