# Correction: Cannabis-based extract for managing pain in dogs with osteoarthritis: efficacy and safety assessment

**DOI:** 10.3389/fphar.2025.1758996

**Published:** 2026-01-20

**Authors:** Neide Maria Griebeler, Ricardo Penayo Cremonese, Yasmin Rafaela Fakih Correa, Priscila Romero Mazzini Pereira, Amanda Furjan Rial, Emanuela Leite, Maria Victoria Luz Gonçalves, Luiz Renato Marques das Almas, Nedice Borges Cardoso, Fernando Cezar-dos-Santos, Aline Theodoro Toci, Andrés Mojoli Le-Quesne, Francisney Pinto Nascimento

**Affiliations:** 1 Laboratory of Cannabis and Psychedelics - LCP, Universidade Federal da Integração Latino Americana (UNILA), Foz do Iguaçu, Brazil; 2 POP-VET Veterinary Hospital, Foz do Iguaçu, Brazil; 3 Associação Santa Cannabis, Florianópolis, Brazil; 4 Environmental and Food Interdisciplinary Studies Laboratory - LEIMAA, Universidade Federal da Integração Latino Americana (UNILA), Foz do Iguaçu, Brazil

**Keywords:** adverse events, cannabinoids, canine, efficacy, osteoarthritis, quality of life, safety

The figure captions were in the wrong order in the PDF/HTML version of this paper. Figure 1 had the caption of Figure 2; Figure 2 had the caption of Figure 1; Figure 3 had the caption of Figure 6; Figure 4 had the caption of Figure 3. The order has now been corrected.

**FIGURE 1:**
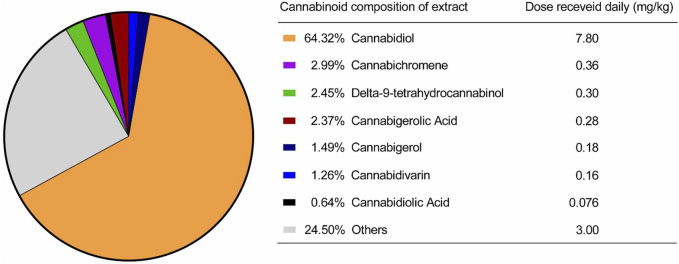
Cannabinoids present in the extract provided by the Santa Cannabis Association.

**FIGURE 2:**
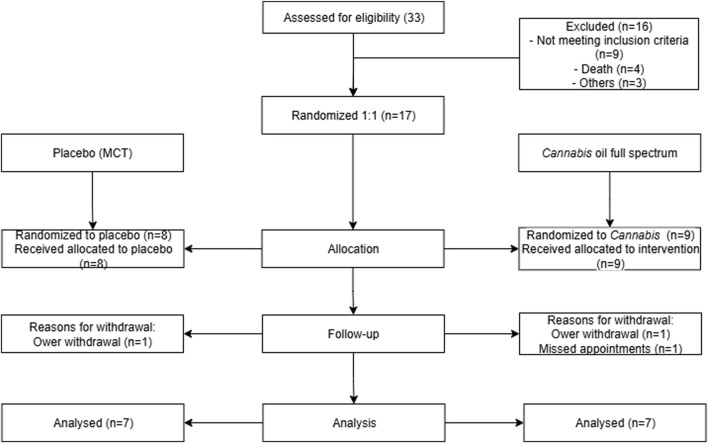
Flowchart of patient triage. This image summarizes the animal selection and study methodology over 90 days, representing the excluded animals and treatment groups.

**FIGURE 3:**
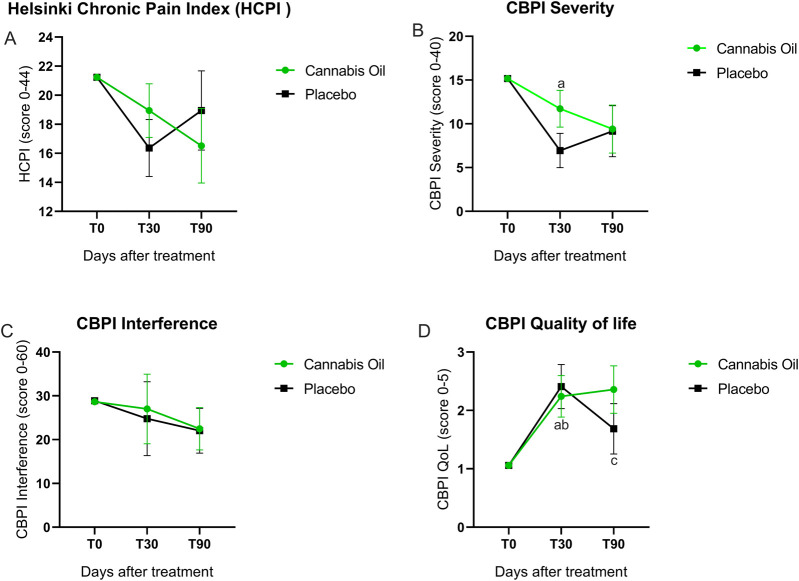
Graphical representation of HCPI and CBPI outcomes.

**FIGURE 4:**
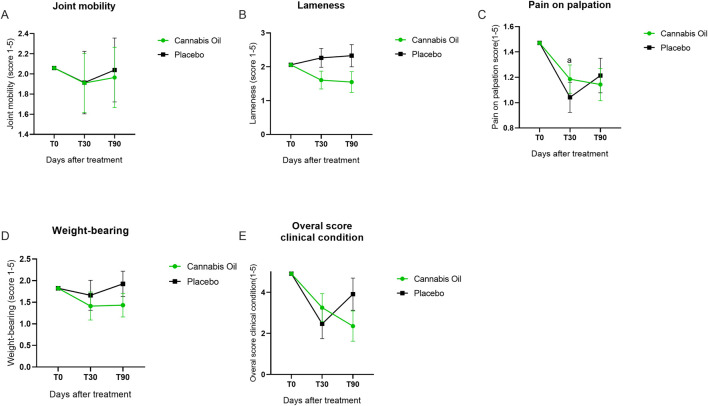
Graphical summary of veterinary clinical assessment outcomes.

Furthermore, there was a mistake in the caption of Figure 5 as published. Figure 5 had the caption of Figure 6. The corrected caption of Figure 5 appears below.

**FIGURE 5:**
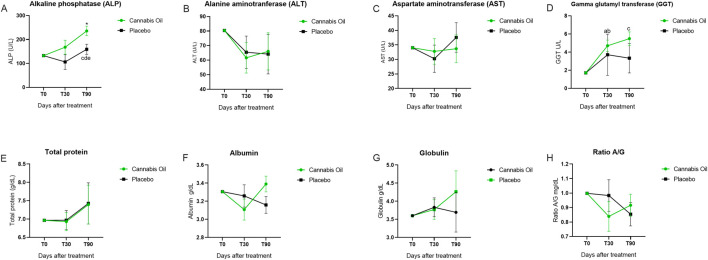
Biochemical marker levels during the 90-day treatment period. Part I. *p < 0.05 for significant differences between cannabis oil and placebo groups. **(A)** p < 0.05 for significant differences between time points 0 and 30 within the placebo group. **(B)** P < 0.05 for significant differences between time points 0 and 30 within the cannabis oil group. **(C)** p < 0.05 for significant differences between time points 0 and 90 within the cannabis oil group. **(D)** p < 0.05 for significant differences between time points 30 and 90 within the cannabis oil group. **(E)** p < 0.05 for significant differences between time points 30 and 90 within the placebo group.

**FIGURE 6:**
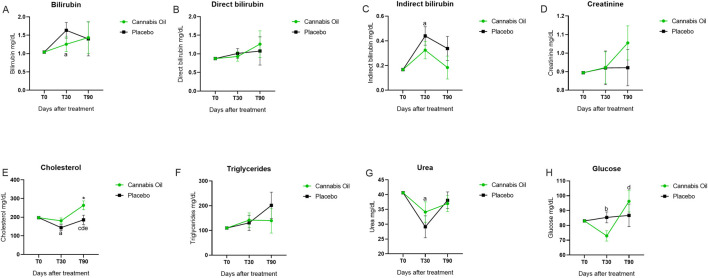
Biochemical marker levels during the 90-day treatment period. Part II.*p < 0.05 for significant differences between cannabis oil and placebo groups. **(A)** P < 0.05 for significant differences between time points 0 and 30 with in the placebo group. **(B)** P < 0.05 for significant differences between time points 0 and 30 with in the cannabis oil group. **(C)** P < 0.05 for significant differences between time points 0 and 90 with in the cannabis oil group. **(D)** P < 0.05 for Q27 significant differences between time points 30 and 90 with in the cannabis oilgroup. **(E)** P < 0.05 for significant differences between time points 30 and 90 with in the placebo group.

There was also a mistake in the caption of Figure 6 as published. Figure 6 had the caption of Figure 3. The corrected caption of Figure 6 appears below.

The original article has been updated.

