# Supplementary figures and images for: Cannabis-based extract for managing pain in dogs with osteoarthritis: efficacy and safety assessment
Source: Front Pharmacol. 2025 Nov 24;16:1539704. doi: 10.3389/fphar.2025.1539704 (PMC12682811; doi:10.3389/fphar.2025.1539704)

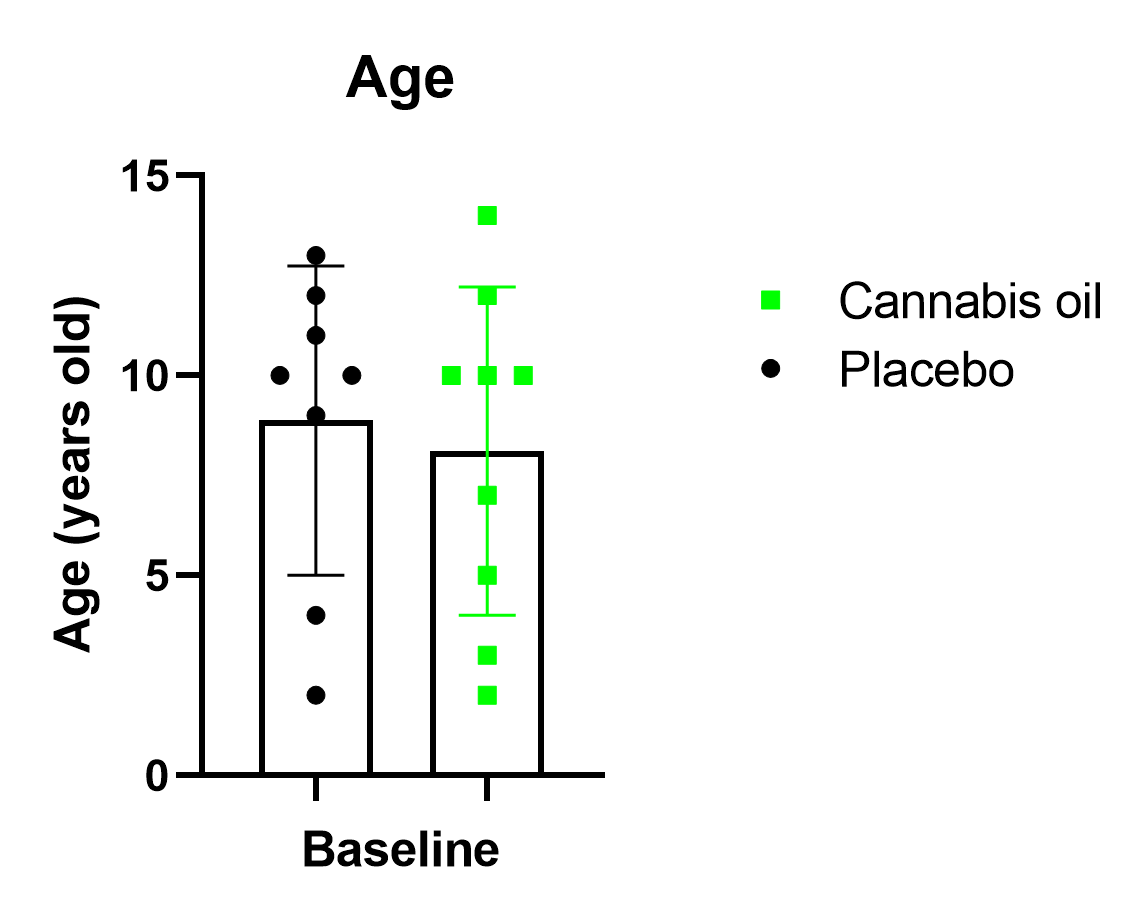

Supplement: Supplementary file 2 [file Image2.tif]

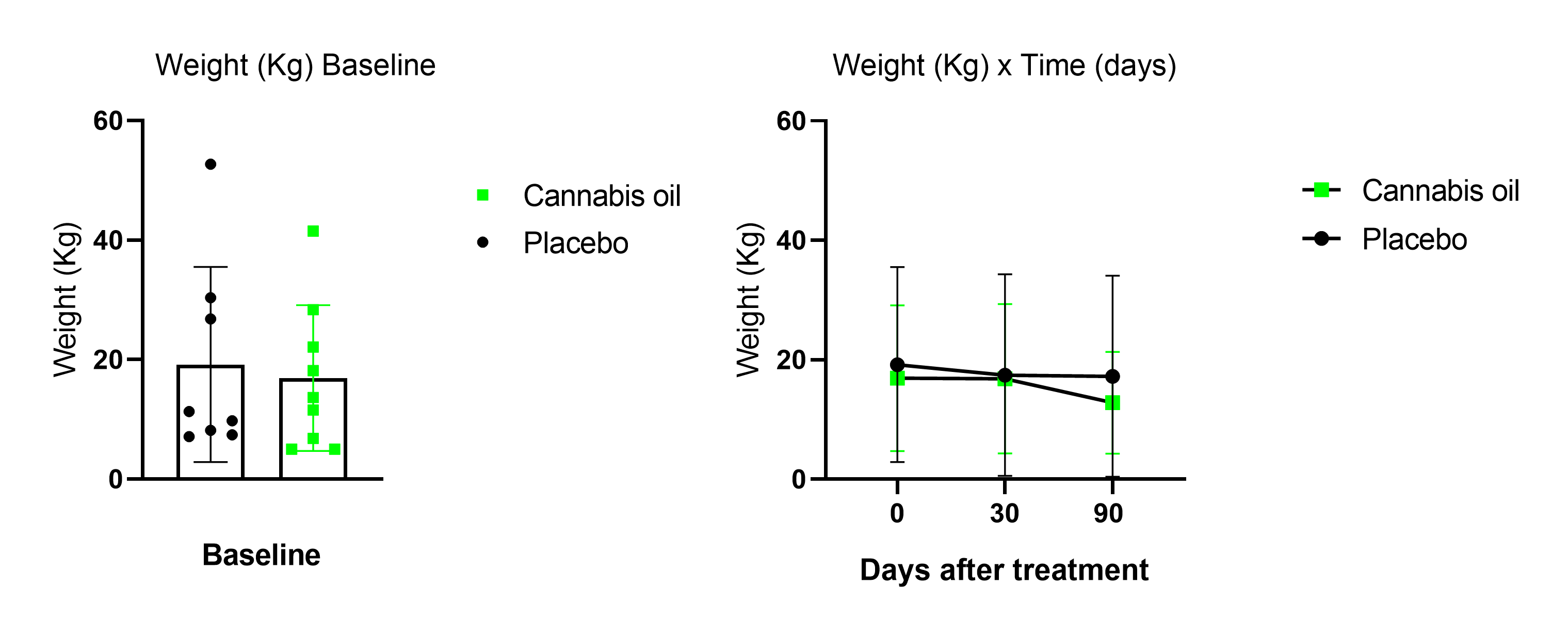

Supplement: Supplementary file 3 [file Image1.tif]
