# Supplementary material for: Cannabis-based extract for managing pain in dogs with osteoarthritis: efficacy and safety assessment
Source: Front Pharmacol. 2025 Nov 24;16:1539704. doi: 10.3389/fphar.2025.1539704 (PMC12682811; doi:10.3389/fphar.2025.1539704)
Supplement: Supplementary file 4 [file DataSheet3.pdf]

Clinical scoring system for assessing dogs with osteoarthritis

Criterion	Clinical evaluation
Lameness	1. Walks normally<br>2. Slightly lame when walking<br>3. Moderately lame when walking<br>4. Severely lame when walking<br>5. Reluctant to rise and will not walk more than five paces
Joint mobility	1. Full range of motion<br>2. Mild limitation (10–20%) in range of motion; no crepitus<br>3. Mild limitation (10–20%) in range of motion; with crepitus<br>4. Moderate limitation (20–50%) in range of motion; ±crepitus<br>5. Severe limitation (>50%) in range of motion; ±crepitus
Pain on palpation	1. None<br>2. Mild signs; dog turns head in recognition<br>3. Moderate signs; dog pulls limb away<br>4. Severe signs; dog vocalises or becomes aggressive<br>5. Dog will not allow palpation
Weight-bearing	1. Equal on all limbs standing and walking<br>2. Normal standing; favours affected limb when walking<br>3. Partial weight-bearing standing and walking<br>4. Partial weight-bearing standing; non-weight-bearing walking<br>5. Non-weight-bearing standing and walking
Overall score of clinical condition	1. Not affected<br>2. Mildly affected<br>3. Moderately affected<br>4. Severely affected<br>5. Very severely affected
