# Supplementary material for: Cannabis-based extract for managing pain in dogs with osteoarthritis: efficacy and safety assessment
Source: Front Pharmacol. 2025 Nov 24;16:1539704. doi: 10.3389/fphar.2025.1539704 (PMC12682811; doi:10.3389/fphar.2025.1539704)
Supplement: Supplementary file 5 [file DataSheet1.pdf]

Canine Brief Pain Inventory

Description of pain:

Rate your dog's pain:

1. Fill in the oval next to the one number that best describes the pain at its **worst** in the last 7days.

○0 ○1 ○2 ○3 ○4 ○5 ○6 ○ 7 ○ 8 ○ 9 ○ 10

No pain Extreme pain

2. Fill in the oval next to the one number that best describes the pain at its **least** in the last 7 days

○0 ○1 ○2 ○3 ○4 ○5 ○6 ○ 7 ○ 8 ○ 9 ○ 10

No pain Extreme pain

3. Fill in the oval next to the one number that best describes the pain at its **average** in the last 7 days.

○0 ○1 ○2 ○3 ○4 ○5 ○6 ○ 7 ○ 8 ○ 9 ○ 10

No pain Extreme pain

4. Fill in the oval next to the one number that best describes the pain as it is **right now**.

○0 ○1 ○2 ○3 ○4 ○5 ○6 ○ 7 ○ 8 ○ 9 ○ 10

No pain Extreme pain

Description of function:

Fill in the oval next to the one number that best describes how during the last 7 days **pain has interfered** with your dog's:

5. **General Activity**

○0 ○1 ○2 ○3 ○4 ○5 ○6 ○ 7 ○ 8 ○ 9 ○ 10

Does not interfere Completely interferes

6. **Enjoyment of Life**

○0 ○1 ○2 ○3 ○4 ○5 ○6 ○ 7 ○ 8 ○ 9 ○ 10

Does not interfere Completely interferes

7. **Ability to Rise to Standing From Lying Down**

○0 ○1 ○2 ○3 ○4 ○5 ○6 ○ 7 ○ 8 ○ 9 ○ 10

Does not interfere Completely interferes

Brief Pain Inventory, con't

8. **Ability to Walk**

○0 ○1 ○2 ○3 ○4 ○5 ○6 ○ 7 ○ 8 ○ 9 ○ 10

Does not interfere — Completely interferes

9. **Ability to Run**

○0 ○1 ○2 ○3 ○4 ○5 ○6 ○ 7 ○ 8 ○ 9 ○ 10

Does not interfere — Completely interferes

10. **Ability to Climb Stairs, Curbs, Doorsteps, etc.**

○0 ○1 ○2 ○3 ○4 ○5 ○6 ○ 7 ○ 8 ○ 9 ○ 10

Does not interfere — Completely interferes

Overall impression:

11. Fill in the oval next to the one number that best describes your dog's overall quality of life over the last 7 days.

○ Poor ○ Fair ○ Good ○ Very Good ○ Excellent
